# Supplementary material for: Investigating the association between educational attainment and allostatic load with risk of cancer mortality among African American women
Source: BMC Womens Health. 2023 Aug 24;23:448. doi: 10.1186/s12905-023-02529-3 (PMC10463695; doi:10.1186/s12905-023-02529-3)
Supplement: Supplementary file 1 — Supplemental Table 1: Unweighted Cox proportional hazard models for the association between educational attainment and allostatic load status with risk of cancer death presented as hazard ratios (HRs) and associated 95% confidence intervals (CIs), among 4,677 non-Hispanic Black women participants with 241 cancer-related deaths. [file 12905_2023_2529_MOESM1_ESM.docx]

| Supplemental Table 1: Unweighted Cox proportional hazard models for the association between educational attainment and allostatic load status with risk of cancer death presented as hazard ratios (HRs) and associated 95% confidence intervals (CIs), among 4,677 non-Hispanic Black women participants with 241 cancer-related deaths. | | | | |
| --- | --- | --- | --- | --- |
|  | **No. (%)**  **Cancer**  **Deaths** | **Hazard Ratio (HR) and 95% Confidence Interval (CI)** | | |
|  |  |  | | |
| **Educational Attainment and Allostatic Load Status** |  | Unadjusted | Age Adjusted | Fully Adjusted |
| College graduate with low allostatic load | 6 (2.3) | 1.00 (Referent) | 1.00 (Referent) | 1.00 (Referent) |
| College graduate with high allostatic load | 12 (4.7) | 1.98 (0.74 – 5.28) | 1.17 (0.44 – 3.14) | 1.04 (0.39 – 2.77) |
| Some college with low allostatic load | 17 (3.2) | 1.34 (0.53 – 3.39) | 1.59 (0.63 – 4.04) | 1.37 (0.53 – 3.51) |
| Some college with high allostatic load | 28 (4.7) | 2.10 (0.87 – 5.07) | 1.40 (0.58 – 3.39) | 1.18 (0.48 – 2.89) |
| HS diploma or equiv. with low allostatic load | 12 (1.8) | 0.67 (0.25 – 1.78) | 0.92 (0.35 – 2.46) | 0.75 (0.28 – 2.05) |
| HS diploma or equiv. with high allostatic load | 55 (6.5) | 2.47 (1.06 – 5.75) | 1.74 (0.75 – 4.05) | 1.40 (0.59 – 3.32) |
| <HS with low allostatic load | 15 (3.2) | 1.33 (0.52 – 3.43) | 1.38 (0.54 – 3.57) | 1.03 (0.39 – 2.72) |
| <HS with high allostatic load | 96 (9.2) | 4.35 (1.91 – 9.92) | 1.79 (0.77 – 4.15) | 1.39 (0.59 – 3.31) |
| **Educational Attainment Stratified Results** | | | | |
| **Among participants with <HS (n = 1,517)** |  |  |  |  |
| Low allostatic load | 15 (3.2) | 1.00 (Referent) | 1.00 (Referent) | 1.00 (Referent) |
| High allostatic load | 96 (9.2) | 3.23 (1.87 – 5.57) | 1.43 (0.80 – 2.55) | 1.43 (0.77 – 2.65) |
| **Among participants with HS Diploma or Equiv. (n = 1,511)** |  |  |  |  |
| Low allostatic load | 12 (1.8) | 1.00 (Referent) | 1.00 (Referent) | 1.00 (Referent) |
| High allostatic load | 55 (6.5) | 3.72 (1.99 – 6.96) | 1.70 (0.88 – 3.29) | 1.70 (0.83 – 3.46) |
| **Among participants with Some College (n = 1,122)** |  |  |  |  |
| Low allostatic load | 17 (3.2) | 1.00 (Referent) | 1.00 (Referent) | 1.00 (Referent) |
| High allostatic load | 28 (4.7) | 1.58 (0.86 – 2.89) | 0.81 (0.42 – 1.54) | 0.83 (0.42 – 1.66) |
| **Among participants with College Graduate or More (n = 514)** |  |  |  |  |
| Low allostatic load | 6 (2.3) | 1.00 (Referent) | 1.00 (Referent) | 1.00 (Referent) |
| High allostatic load | 12 (4.7) | 1.91 (0.72 – 5.10) | 1.14 (0.40 – 3.24) | 1.07 (0.34 – 3.42) |
| p-value for interaction between education and allostatic load | | 0.30 | 0.56 | 0.57 |
| Hazard ratios are estimated using Cox proportional hazard models with sample treated as a simple random sample.  Fully adjusted is for age, family poverty to income ratio, and current smoker status. | | | | |
