# Supplementary material for: Investigating the association between educational attainment and allostatic load with risk of cancer mortality among African American women
Source: BMC Womens Health. 2023 Aug 24;23:448. doi: 10.1186/s12905-023-02529-3 (PMC10463695; doi:10.1186/s12905-023-02529-3)
Supplement: Supplementary file 2 — Supplemental Table 2: Excluding 180 NHANES participants with history of cancer survey, weighted Cox proportional hazard models presented as Hazard Ratios (HR) and 95% Confidence Intervals (CI) for the association between educational attainment/allostatic load and risk of cancer death, among 4,484 (weighted N = 8,994,349) NHANES participants with 204 (weighted n = 327,898) cancer-related deaths. [file 12905_2023_2529_MOESM2_ESM.docx]

| Supplemental Table 2: Excluding 180 NHANES participants with history of cancer survey, weighted Cox proportional hazard models presented as Hazard Ratios (HR) and 95% Confidence Intervals (CI) for the association between educational attainment/allostatic load and risk of cancer death, among 4,484 (weighted *N =* 8,994,349) NHANES participants with 204 (weighted n = 327,898) cancer-related deaths. | | | | | |
| --- | --- | --- | --- | --- | --- |
|  | **No. &**  **(Weighted %)**  **Cancer**  **Deaths** | **Mean Survival**  **Months (SE)** | **Hazard Ratio (HR) and 95% Confidence Interval (CI)** | | |
| **Educational Attainment and Allostatic Load Status** |  |  | Unadjusted | Age Adjusted | Fully Adjusted |
| College graduate or more with low allostatic load | 6 (2.8) | 308.6 (1.9) | 1.00 (Referent) | 1.00 (Referent) | 1.00 (Referent) |
| College graduate or more with high allostatic load | 7 (2.7) | 275.5 (2.0) | 0.95 (0.26 – 3.42) | 0.56 (0.15 – 2.06) | 0.48 (0.13 – 1.75) |
| Some college with low allostatic load | 15 (2.7) | 246.2 (1.2) | 0.93 (0.28 – 3.17) | 1.08 (0.32 – 3.68) | 0.91 (0.27 – 3.12) |
| Some college with high allostatic load | 21 (2.8) | 235.8 (1.2) | 1.06 (0.38 – 2.99) | 0.70 (0.24 – 2.02) | 0.59 (0.20 – 1.68) |
| HS diploma or equiv. with low allostatic load | 10 (1.3) | 260.7 (0.7) | 0.38 (0.14 – 1.07) | 0.47 (0.17 – 1.28) | 0.37 (0.14 – 0.96) |
| HS diploma or equiv. with high allostatic load | 49 (5.5) | 251.8 (1.3) | 1.71 (0.65 – 4.52) | 1.12 (0.43 – 2.87) | 0.87 (0.34 – 2.21) |
| <HS with low allostatic load | 14 (2.3) | 290.7 (1.7) | 0.81 (0.29 – 2.22) | 0.80 (0.29 – 2.22) | 0.57 (0.21 – 1.58) |
| <HS with high allostatic load | 82 (6.6) | 294.8 (2.1) | 2.56 (1.05 – 6.22) | 0.99 (0.39 – 2.51) | 0.74 (0.30 – 1.82) |
| **Educational Attainment Stratified Results** | | | | | |
| **Among participants with <HS (n = 1,447)** |  |  |  |  |  |
| Low allostatic load | 14 (2.3) | 290.7 (1.7) | 1.00 (Referent) | 1.00 (Referent) | 1.00 (Referent) |
| High allostatic load | 82 (6.6) | 294.8 (2.1) | 3.12 (1.76 – 5.53) | 1.31 (0.66 – 2.59) | 1.29 (0.66 – 2.55) |
| **Among participants with HS Diploma or Equiv. (n = 1,455)** |  |  |  |  |  |
| Low allostatic load | 10 (1.3) | 260.7 (0.7) | 1.00 (Referent) | 1.00 (Referent) | 1.00 (Referent) |
| High allostatic load | 49 (5.5) | 251.8 (1.3) | 4.54 (2.11 – 9.74) | 2.27 (1.08 – 4.74) | 2.26 (1.10 – 4.57) |
| **Among participants with Some College (n = 1,074)** |  |  |  |  |  |
| Low allostatic load | 15 (2.7) | 246.2 (1.2) | 1.00 (Referent) | 1.00 (Referent) | 1.00 (Referent) |
| High allostatic load | 21 (2.8) | 235.8 (1.2) | 1.13 (0.46 – 2.83) | 0.59 (0.21 – 1.70) | 0.60 (0.21 – 1.67) |
| **Among participants with College Graduate or More (n = 493)** |  |  |  |  |  |
| Low allostatic load | 6 (2.8) | 308.6 (1.9) | 1.00 (Referent) | 1.00 (Referent) | 1.00 (Referent) |
| High allostatic load | 7 (2.7) | 275.5 (2.0) | 0.94 (0.25 – 3.45) | 0.64 (0.14 – 2.83) | 0.59 (0.12 – 2.99) |
| p-value for interaction between education and allostatic load | | | <0.001 | <0.001 | <0.001 |
| Percentages are weighted. Cox proportional hazard models are estimated using NHANES survey weighting.  Mean survival months are unweighted.  Fully adjusted is for age, family poverty to income ratio, and current smoker status. | | | | | |
